# Continuous improvement through differential trajectories of individual minimal disease activity criteria with guselkumab in active psoriatic arthritis: post hoc analysis of a phase 3, randomized, double-blind, placebo-controlled study

**DOI:** 10.1186/s41927-024-00375-w

**Published:** 2024-02-04

**Authors:** Laura C. Coates, Proton Rahman, Philip J. Mease, May Shawi, Emmanouil Rampakakis, Alexa P. Kollmeier, Xie L. Xu, Soumya D. Chakravarty, Iain B. McInnes, Lai-Shan Tam

**Affiliations:** 1 Nuffield Department of Orthopaedics, Rheumatology and Musculoskeletal Sciences, University of Oxford, Oxford, UK; 2 Craig L Dobbin Genetics Research Centre, St. John’s, Newfoundland and Labrador, Memorial University of Newfoundland, St. John’s, NL Canada; 3 Department of Rheumatology Research, Swedish Medical Center/Providence St Joseph Health, University of Washington, Seattle, WA USA; 4 Janssen Research & Development, LLC, Titusville, NJ USA; 5 Department of Pediatrics, McGill University, Montreal, QC Canada; 6 JSS Medical Research, Inc, Scientific Affairs, Montreal, QC Canada; 7 Janssen Research & Development, LLC. Immunology, San Diego, California USA; 8 Janssen Scientific Affairs, LLC, Horsham, PA USA; 9 Division of Rheumatology, Drexel University College of Medicine, Philadelphia, PA USA; 10 College of Medical Veterinary and Life Sciences, University of Glasgow, Glasgow, UK; 11 Department of Medicine & Therapeutics, The Chinese University of Hong Kong, Sha Tin, Hong Kong China

**Keywords:** Psoriatic arthritis, Biologics, Guselkumab, Minimal disease activity

## Abstract

**Background:**

To explore the trajectory of, and factors contributing to, achievement of individual criteria of minimal disease activity (MDA) in patients with active psoriatic arthritis (PsA) treated with guselkumab.

**Methods:**

The Phase 3, randomized, placebo-controlled DISCOVER-2 study enrolled adults (*N* = 739) with active PsA despite standard therapies who were biologic/Janus kinase inhibitor-naive. Patients were randomized 1:1:1 to guselkumab 100 mg every 4 weeks; guselkumab 100 mg at week 0, week 4, then every 8 weeks; or placebo. In this post hoc analysis, patients randomized to guselkumab were included and pooled (*N* = 493). Longitudinal trajectories of achieving each MDA criterion through week 100 were derived using non-responder imputation. Time to achieve each criterion was estimated with Kaplan-Meier analysis. Multivariate regression for time to achieve each criterion (Cox regression) and achievement at week 100 (logistic regression) was used to identify contributing factors.

**Results:**

Continuous improvement across all MDA domains was shown over time. ~70% of patients achieved near remission in swollen joint count (SJC), Psoriasis Area and Severity Index (PASI), and enthesitis through week 100. Median times to achieve individual criteria differed significantly (*p* < 0.0001), with SJC ≤ 1 (20 weeks), PASI ≤ 1 (16 weeks), and ≤ 1 tender entheses (16 weeks) being faster than patient-reported criteria (pain ≤ 15 mm, patient global assessment of arthritis and psoriasis ≤ 20 mm, Health Assessment Questionnaire-Disability Index ≤ 0.5) and tender joint count ≤ 1. Higher baseline domain scores, older age, worse fatigue, and increased body mass index were significant predictors of longer time to achieve minimal levels of disease activity assessed via patient-reported criteria.

**Conclusions:**

Substantial proportions of guselkumab-treated patients achieved individual MDA criteria, each showing continuous improvement through week 100, although with distinct trajectories. Median times to achieve physician-assessed MDA criteria were significantly faster compared with patient-driven criteria. Identification of modifiable factors affecting the time to achieve patient-reported criteria has the potential to optimize the achievement and sustainability of MDA in the clinic via a multidisciplinary approach to managing PsA, involving both medical and lifestyle interventions.

**Trial registration number:**

NCT03158285.

**Trial registration date:**

May 16, 2017.

## Introduction

Psoriatic arthritis (PsA) is an inflammatory arthritis with significant skin and joint involvement, and heterogeneous clinical presentation including peripheral joint pain, skin and nail lesions, enthesitis, dactylitis, and axial disease [1, 2]. Composite measures historically used to evaluate PsA disease activity were adapted from other disease states (e.g., rheumatoid arthritis) and did not encompass all PsA disease domains [3]. To address this gap, minimal disease activity (MDA) criteria were developed as a composite set of measures evaluating multiple PsA domains. MDA is defined as the fulfillment of ≥ 5 of the following 7 criteria [3]: tender joint count (TJC) ≤ 1, swollen joint count (SJC) ≤ 1, Psoriasis Area and Severity Index (PASI) score ≤ 1 or body surface area (BSA) ≤ 3%, patient-reported pain (Pt Pain) visual analog scale (VAS) ≤ 15 mm, patient global assessment of disease activity (arthritis and psoriasis; PtGA) VAS ≤ 20 mm, Health Assessment Questionnaire-Disability Index (HAQ-DI) [4] score ≤ 0.5, and ≤ 1 tender entheseal points. Since its development, MDA has been validated in PsA across multiple studies [5–8], and its prognostic value has been shown in terms of quality of life, fatigue, and other patient-reported outcomes [6, 9].

Despite the availability of increasingly effective treatments in recent years, a minority of PsA patients achieve MDA and few realize sustained MDA [5, 10]. In a real-world analysis of patients with PsA who received infliximab, golimumab, or ustekinumab, the MDA criteria that were least likely to be achieved were Pt Pain, PtGA, and PASI [5]. Of note, criteria for patient-reported domains of MDA may be less frequently achieved [5, 11], owing to comorbidities associated with systemic inflammatory conditions [12], including fibromyalgia and obesity, that may indirectly contribute to unmet MDA domain criteria.

Guselkumab, a fully human monoclonal antibody targeting the p19 subunit of interleukin (IL)-23, is approved to treat adults with moderate-to-severe psoriasis or active PsA [13]. Guselkumab has been shown to have a favorable safety profile relative to placebo and over time and to be associated with significant symptom improvement across PsA domains [14]. In the Phase 3, randomized, placebo-controlled 1-year DISCOVER-1 and 2-year DISCOVER-2 trials, guselkumab 100 mg administered every 4 weeks (Q4W) or at weeks 0 and 4 and then every 8 weeks (Q8W) was shown to induce early and long-standing improvements in multiple domains of PsA, including peripheral joints, skin, enthesitis, dactylitis, and axial symptoms [15–21]. When pooling data from DISCOVER-1 and DISCOVER-2 and employing non-responder imputation (NRI) for missing data, MDA was achieved by 36% and 31% of patients in the guselkumab Q4W and Q8W groups, respectively, at week 52 [22]. In DISCOVER-2, MDA response rates (NRI) were 34% and 31% (week 52) and 38% and 40% (week 100), respectively, in patients receiving guselkumab Q4W and Q8W; approximately 80% of week 52 MDA responders maintained response at week 100 [21].

Limited data exist on the trajectory of achievement of the individual MDA criteria. Using data through 2 years from the DISCOVER-2 study of patients with active PsA, the objectives of this post hoc analysis were to assess the time to achieve the individual MDA criteria and explore factors associated with trajectory of response in these components among patients treated with guselkumab.

## Methods

### Data source and study design

This post hoc analysis utilized data from guselkumab-randomized patients with active PsA through 2 years of the DISCOVER-2 study (NCT03158285). Details of the study design, inclusion and exclusion criteria, and primary results have been reported [19]. Briefly, DISCOVER-2 was a randomized, double-blind, placebo-controlled, Phase 3 study comprising a placebo-controlled period (week 0–24) and an active treatment period (week 24–100) [19]. Patients were randomized 1:1:1 to receive guselkumab 100 mg Q4W; guselkumab 100 mg at week 0, week 4, and then Q8W; or placebo with crossover to guselkumab 100 mg Q4W at week 24. Patients were permitted to continue stable use of selected concomitant medications (e.g., non-steroidal anti-inflammatory drugs, analgesics, oral corticosteroids, or conventional synthetic disease-modifying antirheumatic drugs).

### Patients

Patients eligible for DISCOVER-2 were adults with a diagnosis of PsA for ≥ 6 months who also met the ClASsification criteria for Psoriatic ARthritis (CASPAR) [23] at screening. Enrolled patients had active PsA (SJC ≥ 5; TJC ≥ 5; C-reactive protein ≥ 0.6 mg/dL); current or documented history of psoriasis; and inadequate response to, or intolerance of, standard non-biologic therapies. All patients were naïve to biologic agents and Janus kinase inhibitors [19]. Patients with a medical history of fibromyalgia (*n* = 3) were not excluded.

### Assessments

Details of DISCOVER-2 efficacy assessments and outcome measures have been reported [19]. The current analysis evaluated achievement of each MDA criterion and overall MDA response through week 100. Independent joint assessors evaluated SJC (0–66) and TJC (0–68), and also determined the number of tender entheses using with the Leeds enthesitis index (LEI; 0–6) [24]. The PASI (0–72) [25] was used to measure the severity and extent of psoriasis; BSA affected by psoriasis was only collected at baseline in DISCOVER-2 and was thus not utilized as an MDA criterion. Patients self-reported their pain (Pt Pain) and PtGA (arthritis and psoriasis), both using a 0–100 mm VAS, as well as the degree to which their physical function was impaired using the HAQ-DI (0–3) [4, 26].

### Data analysis

Patients randomized to guselkumab were included in these analyses. Given that previous analyses showed no meaningful differences in clinical efficacy between guselkumab Q4W and Q8W in this biologic-naïve study cohort [19, 22], data from the two dosing regimens were pooled. The proportions of patients achieving each MDA criterion through week 100 were determined among patients not meeting the respective criterion at baseline. Patients with missing data at any timepoint were imputed using NRI. The time to achieve each MDA domain criterion was assessed with the Kaplan-Meier estimator of the survival function. In addition to MDA achievement using scores deriving from native scales, a secondary analysis of domain scores normalized to a 0–66 scale (corresponding to SJC) was also conducted, setting targets to a normalized score of ≤ 1 to account for differences in scales and rigor of criteria.

Baseline determinants (demographic characteristics, disease characteristics and related conditions, exogenous factors) of time to achieve and achievement at week 100 of patient-reported MDA components (Pt Pain, PtGA, HAQ-DI), which showed distinct trajectories in the Kaplan-Maier analyses, were assessed with multivariate Cox proportional hazard models and logistic regression, respectively. Demographic-related determinants evaluated were age, sex, and race (white vs. Asian). Disease characteristics and related conditions evaluated were baseline levels of the respective variable (Pt Pain, PtGA, HAQ-DI) and fatigue assessed using Functional Assessment of Chronic Illness Therapy-Fatigue Scale (FACIT-Fatigue) [27–29]. Exogenous factors evaluated were body mass index (BMI), medical history of depression, baseline presence of suicidal ideation or non-suicidal self-injurious behavior assessed using the electronic Columbia-Suicide Severity Rating Scale (eC-SSRS) [30], medical history of fibromyalgia, and guselkumab regimen (Q4W vs. Q8W). Two sets of models were derived for each MDA criterion: a saturated model including all covariates and a reduced model using stepwise backward selection of significant variables.

All analyses were performed using the statistical software package SAS 9.4 (Statistical Analysis System, SAS Institute Inc., Cary, NC, USA).

## Results

### Baseline characteristics

Detailed baseline characteristics of the overall DISCOVER-2 population and patient disposition through week 100 have been reported [19, 21]. Among the 739 patients enrolled and treated in DISCOVER-2, 493 were randomized to and received guselkumab (Q4W: 245, Q8W: 248) and were included in this post hoc analysis. At baseline, patients had an approximate mean age of 45 years, PsA duration of 5 years, and active PsA, as reflected by numbers of involved joints, levels of pain, and impaired physical function (Table 1). Baseline characteristics were generally well balanced between the two guselkumab regimens.

**Table 1:** Baseline characteristics of guselkumab-randomized patients

	Guselkumab Q4W (*N* = 245)	Guselkumab Q8W (*N* = 248)	Pooled Guselkumab (*N* = 493)
Age, years	45.9 (11.5)	44.9 (11.9)	45.4 (11.7)
Male sex, n (%)	142 (58%)	129 (52%)	271 (55%)
Race, n (%)			
White Asian	242 (99%) 3 (1%)	240 (97%) 8 (3%)	482 (98%) 11 (2%)
BMI, kg/m^2^ Normal (< 18.5–<25 kg/m^2^), n (%) Overweight (25–<30 kg/m^2^), n (%) Obese (≥ 30 kg/m^2^), n (%)	29.1 (5.9) 66 (26.9%) 83 (33.9%) 96 (39.2%)	28.7 (6.3) 74 (29.8%) 82 (33.1%) 92 (37.1%)	28.9 (6.1) 140 (28.4%) 165 (33.5%) 188 (38.1%)
PsA duration, years	5.5 (5.9)	5.1 (5.5)	5.3 (5.7)
SJC (0–66)	12.9 (7.8)	11.7 (6.8)	12.3 (7.4)
TJC (0–68)	22.4 (13.5)	19.8 (11.9)	21.1 (12.8)
HAQ-DI score (0–3)	1.2 (0.6)	1.3 (0.6)	1.3 (0.6)
PtGA– arthritis VAS (0–10 cm)	6.4 (1.9)	6.5 (1.9)	6.5 (1.9)
Pt Pain VAS (0–10 cm)	6.2 (2.0)	6.3 (2.0)	6.2 (2.0)
PASI score (0–72)	10.8 (11.7)	9.7 (11.7)	10.2 (11.7)
Enthesitis, n (%) LEI score	170 (69%) 3.0 (1.7)	158 (64%) 2.6 (1.5)	328 (67%) 2.8 (1.6)
FACIT-Fatigue score	30.8 (9.6)	29.3 (9.9)	30.0 (9.8)
Medical history of depression, n (%)	4 (1.6%)	4 (1.6%)	8 (1.6%)
Suicidal ideation (eC-SSRS), n (%)*	3 (1.2%)	6 (2.4%)	9 (1.8%)
Medical history of fibromyalgia, n (%)	2 (0.8%)	1 (0.4%)	3 (0.6%)

Data are presented as mean (standard deviation) unless stated otherwise

BMI = body mass index; eC-SSRS = electronic Columbia-Suicidality Severity Rating Scale; FACIT = Functional Assessment of Chronic Illness Therapy; HAQ-DI = Health Assessment Questionnaire-Disability Index; LEI = Leeds enthesitis index; PASI = Psoriasis Area Severity Index; PsA = psoriatic arthritis; Pt = patient; PtGA = patient global assessment; Q4W = every 4 weeks; Q8W = every 8 weeks; SJC = swollen joint count; TJC = tender joint count; VAS = visual analog scale

*Patients with unstable suicidal ideation/behavior who were at risk were excluded from DISCOVER-2

### Proportions of guselkumab-randomized patients achieving MDA criteria

**Fig. 1:**
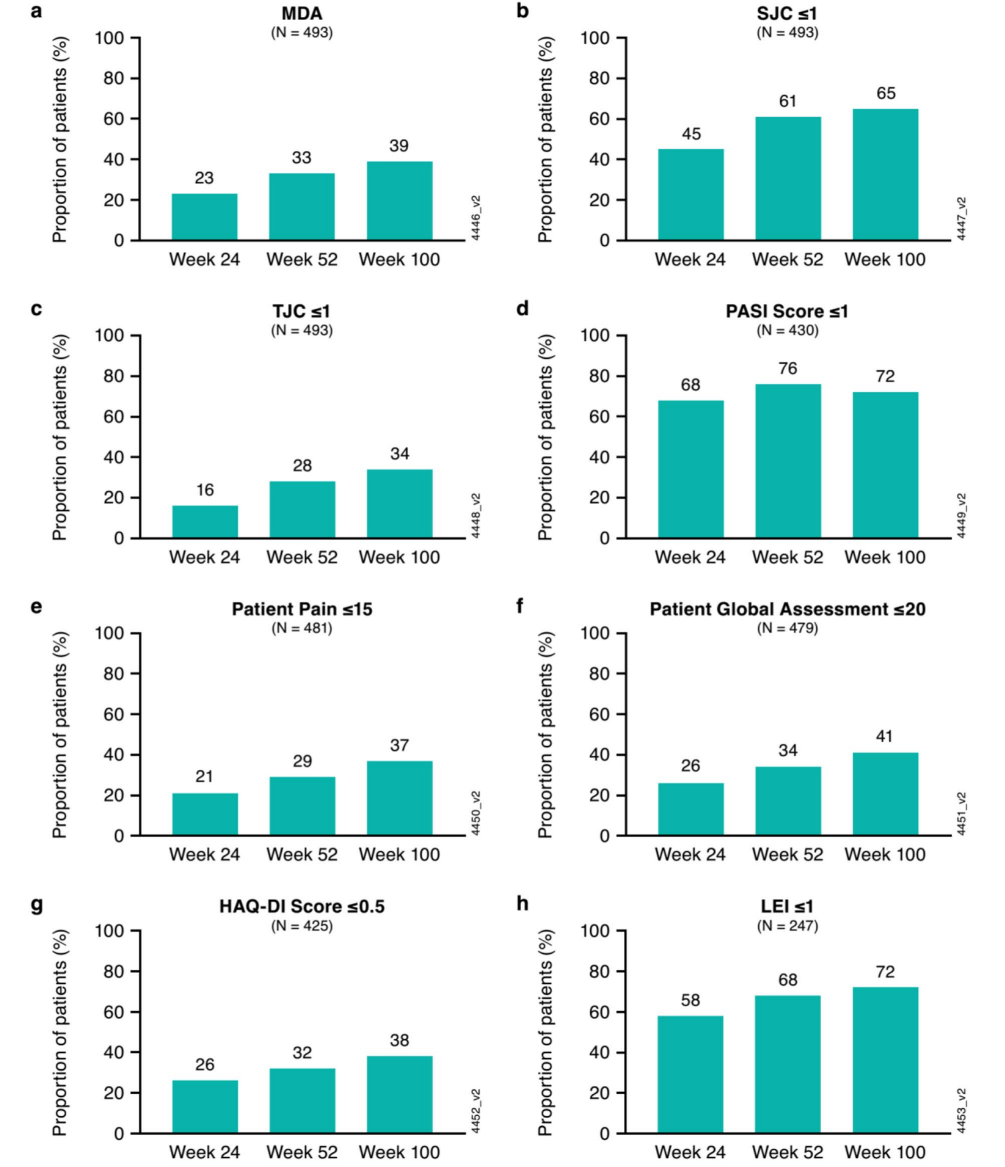
Proportions of guselkumab-randomized patients achieving MDA domain criteria (NRI) at weeks 24, 52, and 100. (**a**) MDA; (**b**) SJC ≤ 1; (**c**) TJC ≤ 1; (**d**) PASI score ≤ 1 among patients with PASI > 1 at baseline; (**e**) Patient pain (Pt Pain) score ≤ 15 mm among patients with Pt Pain > 15 at baseline; (**f**) Patient Global Assessment (PtGA) score ≤ 20 mm among patients with PtGA > 20 mm at baseline; (**g**) HAQ-DI score ≤ 0.5 among patients with HAQ-DI > 0.5 at baseline; (**h**) LEI ≤ 1 among patients with > 1 tender entheses at baseline. HAQ-DI = Health Assessment Questionnaire-Disability Index; LEI = Leeds enthesitis index; MDA = minimal disease activity; NRI = non-responder imputation; PASI = Psoriasis Area Severity Index; SJC = swollen joint count; TJC = tender joint count

Clinical efficacy results of the DISCOVER-2 study through weeks 24, 52, and 100 have been reported [16, 19, 21]. At week 24, the proportion of patients achieving MDA response was significantly higher in the guselkumab Q4W (19%) and guselkumab Q8W (25%) treatment groups compared with placebo (6%) [19]. When evaluated using NRI, increasing proportions of guselkumab-treated patients achieved MDA through week 100 (Fig. 1a), with 23%, 33%, and 39% doing so at weeks 24, 52, and 100, respectively. Consistent patterns were observed in achieving the individual MDA domain criteria through week 100 as shown by the general increase in the proportion of patients achieving each criterion over time (Fig. 1b and h). At 2 years, more than two-thirds of guselkumab-randomized patients achieved minimal joint swelling (SJC ≤ 1), enthesitis (LEI ≤ 1), and skin disease (PASI score ≤ 1). Furthermore, among patients not meeting the respective criteria at baseline, approximately 40% achieved normalized physical function (HAQ-DI score ≤ 0.5) and reported low overall disease activity (PtGA score ≤ 20 mm), while one-third of patients achieved minimal joint tenderness (TJC ≤ 1) and pain (Pt Pain ≤ 15 mm).

### Time to achieve MDA domain criteria

**Fig. 2:**
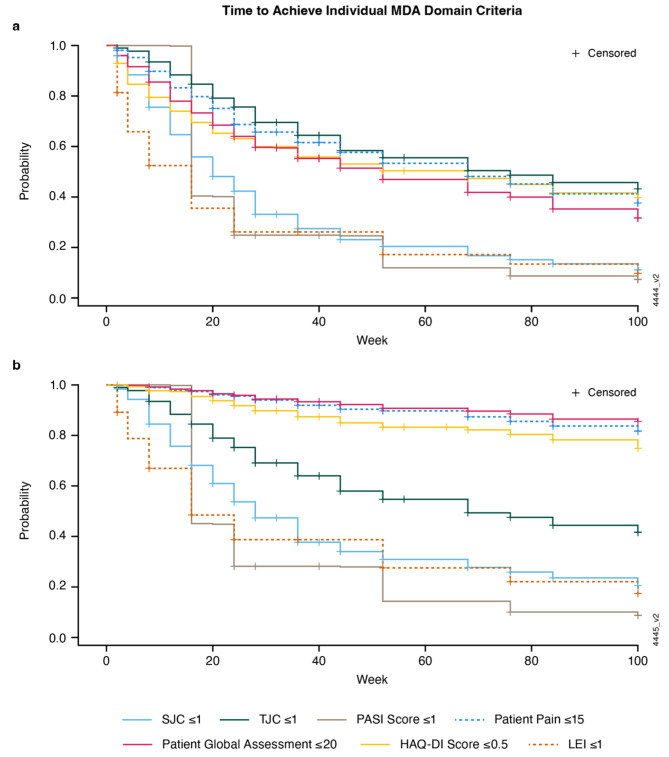
Time to achieve individual MDA domain criteria among guselkumab-randomized patients using scores deriving from native scales (**a**) and scores deriving from normalized scales (**b**). HAQ-DI = Health Assessment Questionnaire-Disability Index; LEI = Leeds enthesitis index; MDA = minimal disease activity; PASI = Psoriasis Area Severity Index; SJC = swollen joint count; TJC = tender joint count

The times to achieve individual MDA criteria with guselkumab differed significantly (*p* < 0.0001), with median times to minimal PASI (16 weeks), enthesitis (16 weeks), and SJC (20 weeks) criteria being faster than for Pt Pain, PtGA, HAQ-DI, and TJC for native-scale scores (Fig. 2a). When normalized both in terms of scale and target score, Pt Pain, PtGA, and HAQ-DI remained the domains with prolonged trajectories (Fig. 2b).

### Determinants of time to achieve criteria for minimal Pt Pain, PtGA, and HAQ-DI

Select baseline variables were identified as significant determinants of time to achieve minimal Pt Pain, PtGA, and HAQ-DI. Following stepwise backward selection of variables entered in multivariate Cox proportional hazard models, higher baseline Pt Pain score (hazard ratio [HR] 0.99, 95% confidence interval [CI] 0.98–1.00; *p* = 0.005), lower baseline FACIT-Fatigue score, indicating more fatigue (HR 1.02, 95% CI 1.00–1.03; *p* = 0.038), and higher BMI (HR 0.98, 95% CI 0.96–1.00; *p* = 0.044) were associated with longer time to achieve minimal pain (Pt Pain ≤ 15 mm). History of fibromyalgia (HR 0.70, 95% CI 0.55–0.90; *p* = 0.004), reported in a small number of patients (*n* = 3), was also associated with longer time to achieve minimal pain (Table 2).

**Table 2:** Determinants of time to achieve Pt Pain ≤ 15 mm and achievement at week 100 among guselkumab-randomized patients (*N* = 493)

Independent baseline variables	Time to Pt Pain ≤ 15 mm achievement		Achievement ofPt Pain ≤ 15 mm at W100	
	HR (95% CI)		OR (95% CI)	
	All covariates	Backward selection	All covariates	Backward selection
Age, years	1.00 (0.99–1.01)	-	1.00 (0.98–1.02)	-
Sex (male vs. female)	1.07 (0.84–1.36)	-	1.09 (0.74–1.61)	-
Race (White vs. Asian)	1.71 (0.52–5.59)	-	1.74 (0.44–6.86)	-
BMI, kg/m^2^	0.98 (0.96–1.00)	**0.98 (0.96–1.00)** ^*^	0.97 (0.94–1.00)^*^	**0.97 (0.94–1.00)** ^*^
Guselkumab treatment (Q4W vs. Q8W)	0.92 (0.73–1.17)	-	0.90 (0.62–1.32)	-
HAQ-DI score (0–3)	-	-	-	-
PtGA VAS (0–10 cm)	-	-	-	-
Pt Pain VAS (0–10 cm)	0.99 (0.98–1.00)^†^	**0.99 (0.98–1.00)** ^**†**^	0.98 (0.97–1.00)^†^	**0.98 (0.97–1.00)** ^**†**^
FACIT-Fatigue^§^	1.02 (1.00–1.03)^*^	**1.02 (1.00–1.03)** ^*^	1.02 (1.00–1.05)	**1.02 (1.00–1.05)** ^*^
History of depression (*N* = 8)	0.86 (0.32–2.32)	-	0.95 (0.22–4.08)	-
Suicidal ideation or non-suicidal self-injurious behaviour (*N* = 9)	0.78 (0.24–2.55)	-	0.49 (0.11–2.12)	-
History of fibromyalgia (*N* = 3)	0.71 (0.56–0.91)^†^	**0.70 (0.55–0.90)** ^**†**^	0.59 (0.40–0.87)^†^	**0.58 (0.40–0.86)** ^**†**^

BMI = body mass index; CI = confidence interval; FACIT = Functional Assessment of Chronic Illness Therapy; HAQ-DI = Health Assessment Questionnaire-Disability Index; HR = hazard ratio; OR = odds ratio; Pt = patient; PtGA = patient global assessment; Q4W = every 4 weeks; Q8W = every 8 weeks; VAS = visual analog scale; W = week

*p < 0.05; ^†^*p* < 0.01

^§^Higher score indicates less fatigue

Worse PtGA score at baseline (HR 0.87, 95% CI 0.81–0.93; *p* < 0.0001) and history of fibromyalgia (HR 0.70, 95% CI 0.55–0.88; *p* = 0.002) were significantly associated with longer time to report PtGA ≤ 20 mm (Table 3). Likewise, higher baseline HAQ-DI score (HR 0.26, 95% CI 0.19–0.36; *p* < 0.0001) and older age (HR 0.98, 95% CI 0.97–0.99; *p* = 0.001) were associated with longer time to achieving normalized physical function (HAQ-DI ≤ 0.5) (Table 4).

**Table 3:** Determinants of time to achieve PtGA ≤ 20 mm and achievement at week 100 among guselkumab-randomized patients (*N* = 493)

Independent baseline variables	Time to PtGA ≤ 20 mm achievement		Achievement of PtGA ≤ 20 mm at W100	
	HR (95% CI)		OR (95% CI)	
	All covariates	Backward selection	All covariates	Backward selection
Age, years	1.00 (0.99–1.01)	-	1.00 (0.98–1.02)	-
Sex (male vs. female)	1.12 (0.89–1.41)	-	1.35 (0.91–2.00)	-
Race (White vs. Asian)	0.85 (0.36–1.97)	-	0.99 (0.24–4.03)	-
BMI, kg/m^2^	0.99 (0.97–1.01)	-	0.98 (0.95–1.02)	-
Guselkumab treatment (Q4W vs. Q8W)	0.96 (0.76–1.21)	-	1.00 (0.68–1.48)	-
HAQ-DI score (0–3)	-	-	-	-
PtGA VAS (0–10 cm)	0.88 (0.82–0.95)^†^	**0.87 (0.81–0.93)** ^**‡**^	0.85 (0.75–0.96)^†^	**0.82 (0.74–0.92)** ^**†**^
Pt Pain VAS (0–10 cm)	-	-	-	-
FACIT-Fatigue^§^	1.01 (1.00–1.03)	-	1.02 (0.99–1.04)	-
History of depression (*N* = 8)	1.36 (0.56–3.33)	-	1.31 (0.30–5.84)	-
Suicidal ideation or non-suicidal self-injurious behaviour (*N* = 9)	1.06 (0.39–2.88)	-	0.57 (0.14–2.40)	-
History of fibromyalgia (*N* = 3)	0.71 (0.56–0.91)^†^	**0.70 (0.55–0.88)** ^**†**^	0.59 (0.39–0.87)^†^	**0.56 (0.38–0.83)** ^**†**^

BMI = body mass index; CI = confidence interval; FACIT = Functional Assessment of Chronic Illness Therapy; HAQ-DI = Health Assessment Questionnaire-Disability Index; HR = hazard ratio; OR = odds ratio; Pt = patient; PtGA = patient global assessment; Q4W = every 4 weeks; Q8W = every 8 weeks; VAS = visual analog scale; W = week

*p < 0.05; ^†^*p* < 0.01; ^‡^*p* ≤ 0.0001

^§^Higher score indicates less fatigue

**Table 4:** Determinants of time to achieve HAQ-DI ≤ 0.5 mm and achievement at week 100 among guselkumab-randomized patients (*N* = 493)

Independent baseline variables	Time to HAQ-DI ≤ 0.5 achievement		Achievement of HAQ-DI ≤ 0.5 at W100	
	HR (95% CI)		OR (95% CI)	
	All covariates	Backward selection	All covariates	Backward selection
Age, years	0.98 (0.97–0.99)^†^	**0.98 (0.97–0.99)** ^**†**^	0.98 (0.96–1.00)^*^	**0.98 (0.96–1.00)** ^*^
Sex (male vs. female)	1.17 (0.90–1.52)	-	1.38 (0.90–2.13)	-
Race (White vs. Asian)	2.36 (0.58–9.66)	-	2.52 (0.50–12.69)	-
BMI, kg/m^2^	0.99 (0.97–1.02)	-	0.98 (0.95–1.02)	-
Guselkumab treatment (Q4W vs., Q8W)	1.01 (0.79–1.30)	-	0.98 (0.64–1.51)	-
HAQ-DI score (0-3)	0.32 (0.23–0.45)^‡^	**0.26 (0.19–0.36)** ^**‡**^	0.16 (0.10–0.27)^‡^	**0.13 (0.08–0.20)** ^**‡**^
PtGA VAS (0–10 cm)	-	-	-	-
Pt Pain VAS (0–10 cm)	-	-	-	-
FACIT-Fatigue^§^	1.01 (1.00–1.03)	-	1.02 (0.99–1.05)	-
History of depression	1.55 (0.64–3.77)	-	1.98 (0.35–11.30)	-
Suicidal ideation or non-suicidal self-injurious behaviour (*N* = 9)	0.20 (0.03–1.42)	-	0.15 (0.03–0.88)^*^	**0.16 (0.03–0.86)** ^*^
History of fibromyalgia (*N* = 3)	0.87 (0.68–1.13)	-	0.84 (0.54–1.30)	-

BMI = body mass index; CI = confidence interval; FACIT = Functional Assessment of Chronic Illness Therapy; HAQ-DI = Health Assessment Questionnaire-Disability Index; HR = hazard ratio; OR = odds ratio; Pt = patient; PtGA = patient global assessment; Q4W = every 4 weeks; Q8W = every 8 weeks; VAS = visual analog scale; W = week

*p < 0.05; ^†^*p* < 0.01; ^‡^*p* ≤ 0.0001

^§^Higher score indicates less fatigue

Patient sex, race, treatment (Q4W vs. Q8W), history of depression, and suicidal ideation or non-suicidal self-injurious behavior were not significantly associated with time to achieve Pt Pain ≤ 15 mm, PtGA ≤ 20 mm, or HAQ-DI ≤ 0.5. Similar results were observed when evaluating the saturated models including all covariates (Tables 2, 3 and 4).

### Determinants of achievement of criteria for minimal Pt pain, PtGA, and HAQ-DI at week 100

In terms of achievement of the individual MDA criteria at week 100, higher baseline Pt Pain score (odds ratio [OR] 0.98, 95% CI 0.97:1.00; *p* = 0.005), lower baseline FACIT-Fatigue score/worse fatigue (OR 1.02, 95% CI 1.00:1.05; *p* = 0.047), higher BMI (OR 0.97, 95% CI 0.94:1.00; *p* = 0.029), and history of fibromyalgia (OR 0.58, 95% CI 0.40:0.86; *p* = 0.007) were associated with lower odds of minimal Pt Pain achievement at week 100 (Table 2).

Worse baseline PtGA score (OR 0.82, 95% CI 0.74:0.92; *p* = 0.0004) and history of fibromyalgia (OR 0.56, 95% CI 0.38:0.83; *p* = 0.004) were associated with non-achievement of PtGA ≤ 20 mm at week 100 (Table 3). Presence of suicidal ideation or non-suicidal self-injurious behavior, reported at baseline by a small number of patients (*n* = 9), was also associated with significantly lower odds of achieving HAQ-DI ≤ 0.5 at week 100 (OR 0.16, 95% CI 0.03:0.86; *p* = 0.033) (Table 4). Older age (OR 0.98, 95% CI 0.96:1.00; *p* = 0.029) and impaired physical function as reflected by baseline HAQ-DI score (OR 0.13, 95% CI 0.08:0.20; *p* < 0.0001) were associated with HAQ-DI ≤ 0.5 non-achievement at week 100. Neither patient sex, race, treatment (Q4W vs. Q8W), PtGA VAS at baseline, nor history of depression impacted the achievement of Pt Pain ≤ 15 mm, PtGA ≤ 20 mm, or HAQ-DI ≤ 0.5. Results were similar in the models including all covariates (Tables 2, 3 and 4).

## Discussion

In this post hoc analysis of the DISCOVER-2 study, which included patients with highly active disease and long disease duration (mean > 5 years), increasing proportions of guselkumab-randomized patients met MDA domain criteria through week 100 based on longitudinal trajectories using NRI. While previous studies have evaluated factors associated with MDA achievement [5, 6, 31] and identified phenotype clusters with different MDA response rates [32], in-depth analyses of the trajectories of individual MDA components and the factors influencing them are necessary to better understand the ‘mechanism’ by which clinical factors affect MDA achievement. To date, only a small Phase 2 study of guselkumab explored individual MDA domains that may be refractory or residual among MDA achievers [18]. A prior analysis of DISCOVER-2 showed an early separation of patients treated with guselkumab from those receiving placebo in achieving improvements across domains considered in the American College of Rheumatology (ACR) response criteria and those shared with MDA, including SJC, TJC, Pt Pain, and HAQ-DI [21]. However, to our knowledge, the trajectory of individual MDA components and time to achievement of response have not been thoroughly investigated.

The present findings agree with analyses demonstrating a long-lasting effect of guselkumab across key PsA domains described by the Group for Research and Assessment of Psoriasis and Psoriatic Arthritis (GRAPPA) [2, 21, 33], along with multi-domain efficacy regardless of baseline demographic and disease characteristics [34]. Findings are also consistent with previous analyses demonstrating the utility of guselkumab in providing stringent and sustained control of disease activity across multiple PsA domains [16, 18, 19, 22, 35, 36], many of which are included in the MDA definition, such as peripheral joint symptoms, psoriatic skin lesions, enthesitis, physical function, and patient pain. Additionally, analysis results reflect the central role of IL-23 in autoimmune inflammation [14] and the unique ability of guselkumab, a fully human IL-23p19-subunit inhibitor [37], to provide continuous overall improvement of PsA signs and symptoms.

Unlike the real-world analysis of patients treated with infliximab, golimumab, or ustekinumab [5], the present study demonstrated that guselkumab-treated patients achieved minimal SJC, PASI, and enthesitis on average within 16–20 weeks, and approximately 70% of patients had achieved near remission in these domains at week 100. The identification of different trajectories of Pt Pain, PtGA, and HAQ-DI in the present analysis is consistent with previous studies demonstrating that patient-reported MDA criteria are achieved less frequently in PsA patients [5, 11]. Furthermore, this finding is also consistent with reports showing that time to achieve low disease activity or remission in patient-reported outcomes was considerably longer compared with physician-assessed criteria, such as SJC in patients with rheumatoid arthritis [38]. This may be explained by differences in the way patients and healthcare providers view disease activity and prioritize different symptoms, and likely also reflects the specificity of outcomes where SJC is typically associated with disease activity while a pain score may be influenced by other factors.

Of the baseline variables evaluated, higher baseline Pt Pain score, greater BMI, and worse fatigue were significant determinants of longer time to achieve minimal Pt Pain and lower probability of achieving minimal Pt Pain at week 100. The observed association between BMI and Pt Pain aligns with two previous studies showing that patients with increased BMI had a lower probability of achieving overall MDA as well as specific criteria, including minimal pain [39, 40]. History of fibromyalgia, which was not an explicit study exclusion criterion but reported in only a small number of patients, was also associated with significantly longer time to achieve Pt Pain ≤ 15 mm. Worse baseline PtGA and history of fibromyalgia were associated with longer time to achieve PtGA ≤ 20 mm and non-achievement at week 100. Older age and higher baseline HAQ-DI score negatively associated with time to report normalized physical function and achievement of HAQ-DI ≤ 0.5 at week 100; these results suggest potential benefits of earlier treatment with biologics and highlight that HAQ-DI scores reflect aging versus lack of disease control. Presence of suicidal ideation/non-suicidal self-injurious behavior at baseline, reported by a small number of patients, was associated with significantly lower odds of achieving HAQ-DI ≤ 0.5 at week 100. It follows that proper care and interdisciplinary intervention for the management of modifiable factors such as pain, fatigue, and mental health may translate to an increased likelihood of achieving MDA, and possibly sustained MDA. Our findings are consistent with previous studies reporting an association between HAQ-DI scores [6] and mental health status [31], including depression and anxiety, and MDA achievement, and provide further insight into the specific domains that are affected by the identified predictors.

MDA is designed to define a state that is a useful target to both patients and clinicians. Although important for predicting patient response to treatment, the ability to predict overall MDA achievement does not provide insight as to the type of intervention that may be needed to optimize patient outcomes. However, predicting longitudinal trajectories of the individual MDA components could form the basis for precision medicine in PsA. Whilst a possible optimization of immunomodulatory therapy should be considered for those patients not in MDA, this is not always an appropriate course of action. It is important in practice to consider which components of MDA are not met and adjust holistic treatment plans accordingly. As mentioned above, this may include other interventions to improve pain or psychological health or to assist with weight loss in overweight patients.

The analyses reported here should be interpreted in light of the known limitations of post hoc analyses, including the potential “cherry picking” of data and lack of statistical power [41]. To that end, it should be noted that a statistical analysis plan was prepared specifically to explore factors associated with trajectory of response for MDA components in patients with PsA receiving treatment with guselkumab prior to conducting any analyses, and that certain baseline factors, despite a potential association with MDA domain criteria achievement, may have not been identified as statistically significant. It is also possible that the median time to achieve minimal PASI was overestimated given that it was not assessed prior to week 16. Furthermore, these results, particularly those observed in patient subgroups with small sample sizes (e.g., patients with history of fibromyalgia, depression, or suicidal ideation), may not be generalizable to real-world populations of patients with PsA and should be interpreted with caution. Finally, the findings of the current analysis may not be generalizable to patients treated with different medications or non-biologic therapies.

In summary, in biologic-naive patients with active PsA from the DISCOVER-2 study, treatment with guselkumab was associated with continuously increasing rates of achieving overall MDA and individual MDA criteria through 2 years. Times to near remission in physician-assessed MDA domains (SJC, PASI, enthesitis) supported rapid achievement of treatment targets with guselkumab. Regarding the patient-reported domains, several determinants were identified including modifiable factors such as BMI, pain, fatigue, and mental health which, with early multidisciplinary management, could assist in optimizing achievement of MDA and possibly sustained MDA.

## Data Availability

The data sharing policy of Janssen Pharmaceutical Companies of Johnson & Johnson is available at: https://www.janssen.com/clinical-trials/transparency. As noted on this site, requests for access to the study data can be submitted through the Yale Open Data Access (YODA) Project site at: http://yoda.yale.edu.

## Abbreviations

BMI: Body mass index
BSA: Body surface area
CASPAR: ClASsification criteria for Psoriatic ARthritis
CI: Confidence interval
eC-SSRS: Electronic Columbia-Suicidality Severity Rating Scale
FACIT: Functional Assessment of Chronic Illness Therapy
GRAPPA: Group for Research and Assessment of Psoriasis and Psoriatic Arthritis
HAQ-DI: Health Assessment Questionnaire-Disability Index
IL: Interleukin
HR: Hazard ratio
LEI: Leeds enthesitis index
MDA: Minimal disease activity
NRI: Non-responder imputation
OR: Odds ratio
PASI: Psoriasis Area and Severity Index
PsA: Psoriatic arthritis
PtGA: Patient global assessment of disease activity
Pt Pain: Patient pain
Q4W: Every 4 weeks
Q8W: Every 8 weeks
SJC: Swollen joint count
TJC: Tender joint count
VAS: Visual analog scale
